# IMPLEMENTATION OF A VIRTUAL FALL PREVENTION PROGRAM IN OLDER VETERANS

**DOI:** 10.1093/geroni/igae098.0708

**Published:** 2024-12-31

**Authors:** Katherine Ritchey

**Affiliations:** VA Puget Sound Healthcare System, Seattle, Washington, United States

## Abstract

Though virtual platforms have increased in popularity few studies have explored the implementation and benefit of a virtually-based fall prevention program. MOVing FREEly (Multicomponent Otago Virtual Falls Reduction Exercise and Education program) is a VA-based fall prevention program delivered entirely over a virtual care platform. The MOVing FREEly program is six-weeks long and includes both education in fall-risk reduction and evidence-based fall prevention exercises. Process measures captured implementation considerations, feasibility and effectiveness of the program. 27 participants who were older (mean 75 years), mostly male (89.0%), white (85.2%) and moderate or high risk of falling completed the program. 70% were new to the virtual platform. Attendance for education and exercise classes was > 80% with little attrition. There was improvement in the Falls Efficacy Scale-International short form and statistically significant improvement in 30 second sit-to-stand and single leg balance tests. Process measures indicated that 40% of participants needed extra help at the start of each class to sign-on to the virtual visit, which was provided by program facilitators. By the fourth class, all participants were self-sufficient in their ability to navigate the virtual platform. Additional considerations such as electronic health record documentation and coding; clinic setup, referral management and virtual appointment scheduling; patient privacy and health emergency planning were necessary for the start-up and sustainability of the program. Virtual fall prevention programs are feasible and effective but require careful logistical planning and considerations. Implementation considerations from this program can guide future telerehabilitation research and clinical efforts.

